# Preoperative motor deficits and depressive symptoms predict quality of life in patients with Parkinson’s disease at different time points after surgery for subthalamic stimulation: a retrospective study

**DOI:** 10.1186/s42466-023-00303-2

**Published:** 2024-02-08

**Authors:** Carolin Semmler, Vasilija Stopic, Stefanie T. Jost, Gereon R. Fink, Peter H. Weiss, Michael T. Barbe

**Affiliations:** 1 Faculty of Medicine, University of Cologne, Cologne, Germany; 2 Department of Neurology, University Hospital Cologne, Cologne, Germany; 3 Cognitive Neuroscience, Institute of Neuroscience and Medicine (INM-3), Forschungszentrum Jülich, Jülich, Germany

**Keywords:** Deep brain stimulation, Subthalamic nucleus, Cognition, Affect, Motor fluctuation

## Abstract

**Background:**

While subthalamic nucleus deep brain stimulation (STN-DBS) improves the quality of life (QoL) of patients with Parkinson’s disease (PD), the clinical parameters that predict this improvement remain debated. This retrospective study explored whether preoperative motor, cognitive, and affective parameters predict QoL or its components at 6 and 12 months after STN-DBS surgery.

**Methods:**

QoL was assessed with the Parkinson’s Disease Questionnaire-39 (PDQ-39) before (baseline), at 6 months (N = 90) and 12 months (N = 63) after STN-DBS surgery. Changes in the PDQ-39 and its subdomains were analysed with Wilcoxon signed-rank tests. In total, seven motor, cognitive, and affective parameters recorded at baseline were used in multiple linear regressions to predict QoL and its subdomains.

**Results:**

QoL had improved significantly at six months post STN-DBS surgery. After 12 months, this effect remained significant but was less pronounced. At both time points, significant improvements in mobility, activities of daily living, stigma, and bodily discomfort were present. Correlation and linear regression analyses showed that preoperative QoL status and changes in QoL at 6 and 12 months after surgery were driven by preoperative dopaminergic medication, as well as motor (UPDRS-III medOFF and PIGD-subscore medOFF) and affective (HADS anxiety and depression) symptoms. In contrast, preoperative cognitive performance did not predict QoL at any time point.

**Conclusion:**

Data show that preoperative motor and affective symptoms drive both QoL baseline status and changes in QoL after STN-DBS surgery. Thus, these clinical parameters need to be assessed appropriately to provide comprehensive presurgical advice to patients suffering from PD.

**Supplementary Information:**

The online version contains supplementary material available at 10.1186/s42466-023-00303-2.

## Highlights

- Quality of life (QoL) improves significantly after STN-DBS surgery in PD patients.
- Cognitive performance does not predict QoL before or after STN-DBS surgery.
- In contrast, pre-operative motor and affective symptoms predict QoL after STN-DBS surgery.
- Prediction strength depends on the time point of postsurgical QoL assessment.
- Depressive symptoms at baseline are associated with changes in QoL after STN-DBS surgery.

**Supplementary Information:**

The online version contains supplementary material available at 10.1186/s42466-023-00303-2.

## Background

With the progression of Parkinson’s disease (PD), its impact on the patients' quality of life (QoL) significantly increases [1]. However, especially in advanced stages of PD, deep brain stimulation of the subthalamic nucleus (STN-DBS) is a well-established neurosurgical treatment modulating disordered basal ganglia activity [2], leading to attenuation of motor symptoms, including fluctuations and dyskinesia [3].

Moreover, STN-DBS benefits PD patients’ QoL [4]. In a meta-analysis, Lachenmayer and colleagues [5] explored 11 studies investigating changes in patients’ QoL 6–24 months after receiving STN-DBS. In all included studies, the patients’ QoL significantly improved [5]. When observing QoL changes after DBS surgery over time, the most substantial improvements were typically achieved within the first 5–6 months, which then slowly regressed with disease progression [6, 7]. Approximately five years after STN-DBS surgery, the PD patients’ QoL eventually returned to the presurgical level [6].

However, when dividing the available studies’ cohorts into responders (i.e., patients who respond to STN-DBS surgery with an improvement in QoL assessed by the Parkinson’s Disease Questionnaire-39 (PDQ-39)) and non-responders (i.e., patients who respond to DBS surgery with no change or a decrease in QoL assessed by the PDQ-39), the latter group represents a relevant part of the total cohort already shortly after surgery. At 6 months post-surgery, the proportion of non-responders is around 43% [8] and increases to 55% after 12 months [9] and to 61% at 36 months after DBS surgery [10]. Although the growth in non-responders over time can be explained by the disease progression, the observation that almost half of the PD patients show no significant QoL improvement 6 months after surgery calls for further refinements of prognostic tools for the QoL response to STN-DBS.

The preoperative QoL status best predicted QoL after STN-DBS [6, 9, 11, 12]. Moreover, a younger age of patients and milder motor symptoms [6] and a lower levodopa equivalent dose (LEDD) [11] before STN-DBS surgery benefit postsurgical QoL outcome [10].

However, further clinical parameters are discussed controversially [9, 11, 12]. The selection of parameters for the prediction analyses may cause these previous ambiguous findings. Although most symptoms in PD can be assigned to three domains [13], motor, cognitive, and affective symptoms are rarely considered together in a mutual analysis, so their relative contributions remain unexplored. The respective methods applied for the regression analysis may also have led to equivocal findings since the often-used exploratory stepwise linear regression chooses variables based on their isolated, individual significance for the chosen sample [14]. This approach ignores the covariance between potential predictors but may also lead to the prioritisation of nuisance variables over true ones and, therefore, to poor out-of-sample accuracy [14]. Another cause for the mixed findings from previous studies might be the variable time points of QoL assessment after DBS surgery. It is conceivable that the impact of pre-surgical predictors changes over time. Therefore, QoL assessments should be performed post-surgically at multiple times to evaluate a given parameter’s time-dependent predictive power. Lastly, previous studies focused solely on the QoL prediction but not its subdomains. However, these cover different essential areas of the patients’ daily lives. The prediction of the QoL subdomains can provide more detailed insights into the influence of pre-surgical parameters on the patients’ QoL after DBS surgery and enables the consideration of interindividual differences between patients’ QoL before surgery.

Therefore, our study investigated the impact of PD patients’ motor, cognitive and affective symptoms on the longitudinal prediction of their QoL up to 12 months after DBS surgery.

## Methods

### Participants and study design

The patients included in this retrospective single-centre longitudinal study were diagnosed with PD according to the UK Brain Bank criteria [15] and screened for DBS treatment according to guidelines by the International Parkinson and Movement Disorder Society [16]. Patients were included in the study, if they were diagnosed with idiopathic Parkinson’s disease, aged 18–90 years and had received surgery for deep brain stimulation of the STN at the university hospital Cologne. Patients were excluded when presenting symptoms of dementia at baseline (PANDA < 14 [17] or MoCA < 20 [18]). Further exclusion criteria were a diagnosis of atypical Parkinson’s disease as well as symptoms of clinically relevant severe depression (as assessed with the BDI-II [19] and the HADS [20]). The study population consisted of 90 patients (62 male) with a mean age of 62.4 years (SD = 8.4). Further clinical characteristics of the study sample are reported in Table 1 and details on the patients non-levodopa medication can be found in Additional file 1: Table S1. All patients underwent surgery for STN-DBS. The data used in this study were acquired during the patient’s clinical care, i.e., during clinical assessments of the patient’s eligibility for DBS and at clinical assessments up to 12 months after surgery. Data were collected and managed using REDCap (Research Electronic Data Capture), a secure, web-based software platform supporting data capture for research studies hosted at the University Hospital Cologne [21, 22]. The study was conducted under the Declaration of Helsinki and authorised by the local ethics committee (Cologne, Study No. 22-1086-retro).

**Table 1:** Baseline clinical characteristics of the PD patients

	N = 90	
Age in years	M (SD)	62.4 (8.4)
Disease duration in years	M (SD)	9.8 (4.9)
Sex		
Female	n (%)	28 (31)
Male	n (%)	62 (69)
LEDD in mg	M (SD)	1068.2 (419.0)
H&Y stage medOFF	Md (IQR)	2.8 (2.5–4.0)
H&Y stage 1	n (%)	0 (0)
H&Y stage 1.5	n (%)	0 (0)
H&Y stage 2	n (%)	20 (22)
H&Y stage 2.5	n (%)	25 (28)
H&Y stage 3	n (%)	22 (24)
H&Y stage 4	n (%)	21 (23)
H&Y stage 5	n (%)	2 (2)
UPDRS-III medOFF	M (SD)	37.0 (11.0)
UPDRS-III medOFF PIGD	Md (IQR)	4.8 (2.8–6.3)
Levodopa Response in %	M (SD)	48.1 (15.8)
MoCA Total	Md (IQR)	25.0 (24.0–27.0)
PANDA Total	Md (IQR)	22.0 (18.0–26.0)
HADS Anxiety T-values	Md (IQR)	49.0 (42.6–55.9)
HADS Depression T-values	Md (IQR)	48.9 (45.3–56.3)
PDQ-39 SI	Md (IQR)	24.0 (14.4–35.6)

*HADS* Hospital Anxiety and Depression Scale, *H&Y medOFF* Hoehn & Yahr assessed without PD-related medication, *IQR* interquartile range, *LEDD* Levodopa equivalent daily dose, *MoCA* Montreal Cognitive Assessment, *PANDA* Parkinson’s Neuropsychometric Dementia Assessment, *PD* Parkinson’s disease, *PDQ-39* Parkinson’s Disease Questionnaire 39, *UPDRS-III medOFF* Unified Parkinson’s Disease Rating Scale Part III assessed without PD-related medication, *UPDRS-III medOFF PIGD* postural instability and gait disorder subscore calculated from item 27–30 of the Unified Parkinson’s Disease Rating Scale Part III assessed without PD-related medication

### Clinical assessment

All patients were assessed using the following questionnaires and tests:

i. QoL was assessed before and at 6 and 12 months after the DBS surgery using the PDQ-39, subsequently reported as the summary index (SI), ranging from 0 (no impairment) to 100 (maximum impairment) [23]. The scale contains motor and non-motor symptoms represented in eight subdomains (mobility, ADL, emotional well-being, stigma, social support, cognition, communication, and bodily discomfort) and is commonly used to assess QoL in patients with PD [5, 8].
ii. Baseline motor symptoms were assessed in the medication OFF state using the Unified Parkinson’s Disease Rating Scale part 3 (UPDRS-III medOFF), ranging from 0 (no impairment) to 108 (maximum impairment), and its subscore for postural instability and gait disorder (PIGD), ranging from 0 (no impairment) to 16 (maximum impairment). Furthermore, the levodopa response was assessed, which is the difference in patient’s motor functions with and without levodopa medication measured using the UPDRS-III and reported as the percentage change.
iii. Baseline cognitive performance was assessed using the Montreal Cognitive Assessment (MoCA) [18] and Parkinson Neuropsychometric Dementia Assessment (PANDA) [17], both ranging from 0 (minimum performance) to 30 (maximum performance).
iv. Baseline affective symptoms were measured using the Hospital Anxiety and Depression Scale (HADS anxiety + depression) [20]. We report the T-values of the HADS scores, ranging from 0 (no symptoms) to 80 (maximum symptoms).
v. Regarding the PD-related medication of the patients, the levodopa equivalent daily dose (LEDD) at the time of the baseline assessment was calculated according to Tomlinson and colleagues [24].

### Statistical analysis

For QoL at baseline (before DBS surgery), the absolute PDQ-39 SI values were used. The significant longitudinal outcome changes in QoL after STN-DBS surgery were computed relative to baseline values. Therefore, follow-up performance at 6 and 12 months after surgery was assessed based on change scores for the total score and each of the eight subscales. Change scores were calculated by subtracting the PDQ-39 SI_Follow-up_ from the PDQ-39 SI_Baseline_. All statistical analyses were performed with SPSS Statistics 28, and the significance level of α = 0.05 was applied unless stated otherwise. The Shapiro–Wilk test was used to check the normal distribution of the clinical data. Data were evaluated using the Wilcoxon signed-rank test since the criteria for parametric testing were not fulfilled. The significance level of the results was corrected for multiple testing per Bonferroni correction. Using Spearman correlations, the relationship between baseline motor, cognitive, and affective candidate predictors and QoL at baseline and follow-up was explored. Then, candidate predictor variables, for which a significant correlation with QoL was found, were used in multiple linear regression to explore the prediction of QoL at baseline and following STN-DBS surgery by these baseline motor, cognitive, and affective parameters. Multicollinearity (r > 0.6) and variance inflation factors (VIF < 10) were checked between all candidate predictor variables.

To further examine a potential relationship between the PD-related medication and the QoL, especially the emotional well-being in patients, correlations between the dosages of the respective medication and the PDQ-39 scores at baseline as well as the change scores after 6 and 12 months after DBS surgery were investigated. Also, a putative relationship between dopaminergic medication and self-reported anxiety and depressive symptoms at baseline was investigated by correlating the dosages of PD-related medication and scores in the HADS anxiety and depression scale.

Lastly, it was checked whether the gender of patients would moderate the prediction effects between the baseline predictors and QoL at baseline or changes in QoL at 6- or 12-months follow-up. For the moderation analysis, the PROCESS macro for SPSS [25] was used.

## Results

### Clinical outcomes

**Fig. 1:**
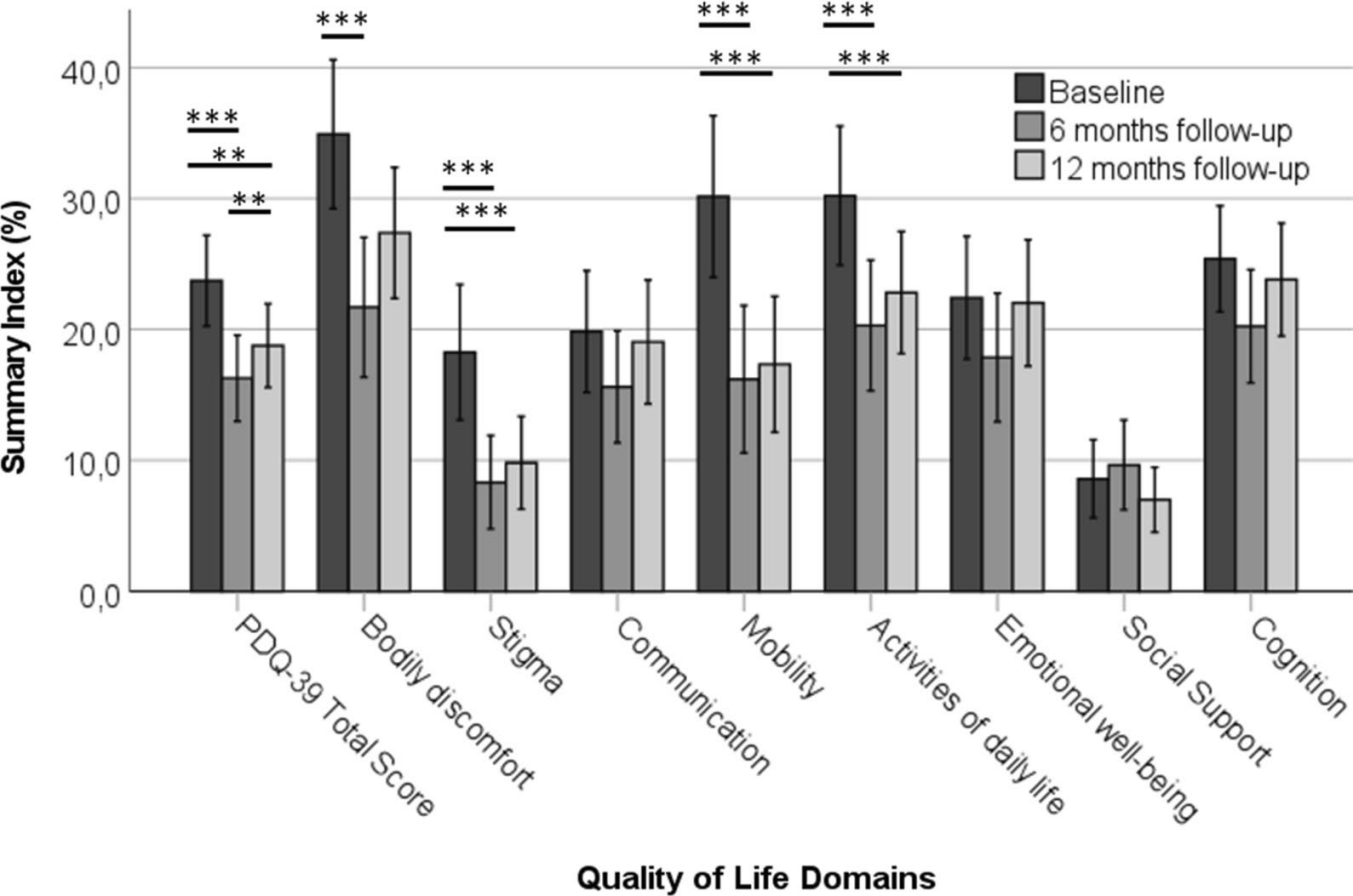
Quality of life at baseline, 6 and 12 months follow-up. The quality of life, displayed as the average summary index (SI) of the Parkinson ‘s Disease Questionnaire 39 (PDQ-39) total and all eight subscales, improved significantly from baseline to 6 and 12 months. The total score slightly but significantly worsened from 6 to 12 months. * = *p* < 0.05, **p < 0.01, ***p < 0.001, PDQ-39 = Parkinson’s Disease Questionnaire 39

The Wilcoxon signed-rank tests revealed that the PDQ-39 total score and the sub-scores for mobility, ADL, stigma and bodily discomfort improved significantly from baseline to 6 months follow-up. Although the total score worsened slightly, albeit significantly from 6 to 12 months follow-up (*p* < 0.01), it was still significantly improved compared to baseline (all *p* < 0.01; Fig. 1). In contrast, the subscales for emotional well-being, social support, cognition, and communication did not significantly change after DBS surgery and almost returned to baseline levels at 12 months follow-up. Notably, compared to the pre-surgery baseline, the reduction in the PDQ-39 total score at 6 and 12 months (− 7.7 and − 6.0 points, respectively) represents meaningful clinical changes (cut off: 4.7 points) [26].

### Correlation analyses

Additional file 1: Tables S2 to S4 provide an overview of all correlations between baseline predictors and patients’ QoL. For clarity, only significant correlations were reported in the following as they were the basis for subsequent regression analyses. Regarding the patients’ QoL at baseline, significant correlations were found between the PANDA total score and the PDQ-39 stigma subscale (r = −0.22, *p* < 0.05) as well as between the LEDD and the PDQ-39 mobility subscale (r = 0.21, *p* < 0.05). Furthermore, the UPDRS-III total score significantly correlated with the PDQ-39 total score as well as the mobility and ADL subscales (r = 0.22, *p* < 0.05; r = 0.34, *p* < 0.001 and r = 0.39, *p* < 0.001, respectively). Also, the UPDRS-III PIGD subscore significantly correlated with the PDQ-39 subscale mobility (r = 0.56, *p* < 0.001), ADL (r = 0.32, *p* < 0.01), emotional well-being (r = 0.25, *p* < 0.05), and the PDQ-39 total score (r = 0.35, *p* < 0.001). Further significant correlations were found between the HADS anxiety score at baseline and the PDQ-39 mobility (r = 0.26, *p* < 0.05), ADL (r = 0.21, *p* < 0.05), emotional well-being (r = 0.40, *p* < 0.001), stigma (r = 0.36, *p* < 0.001), social support (r = 0.26, *p* < 0.01), cognition (r = 0.33, *p* < 0.001), communication (r = 0.43, *p* < 0.001), and bodily discomfort subscale (r = 0.34, *p* < 0.001), and the PDQ-39 total score (r = 0.43, *p* < 0.001) at baseline. Significant correlations between the HADS depression score at baseline were found with the PDQ-39 subscales mobility (r = 0.34, *p* < 0.001), ADL (r = 0.23, *p* < 0.05), emotional well-being (r = 0.47, *p* < 0.001), stigma (r = 0.40, *p* < 0.001), cognition (r = 0.38, *p* < 0.001), communication (r = 0.43, *p* < 0.001), bodily discomfort (r = 0.28, *p* < 0.01), and the PDQ-39 total score (r = 0.43, *p* < 0.001) at baseline.

Regarding QoL at 6 months follow-up, significant correlations were found between the LEDD at baseline and the PDQ-39 stigma change scores (r = −0.23, *p* < 0.05) as well as between the UPDRS-III PIGD subscore at baseline and the PDQ-39 subscale mobility change score (r = 0.24, *p* < 0.05). Also, the HADS anxiety at baseline significantly correlated with the PDQ-39 stigma change scores (r = 0.24, *p* < 0.05). Further significant correlations were found between the HADS depression score at baseline and the change scores of the PDQ-39 stigma subscale (r = 0.27, *p* < 0.05) and PDQ-39 total (r = 0.26, *p* < 0.05).

Regarding QoL at 12 months follow-up, significant correlations were found between the UPDRS-III and the PDQ-39 social support change scores (r = 0.28, *p* < 0.05). Furthermore, the HADS depression scale at baseline significantly correlated with the change scores of the PDQ-39 subscales emotional well-being (r = 0.29, *p* < 0.05), stigma (r = 0.27, *p* < 0.05) and communication (r = 0.32, *p* < 0.01) as well as with the PDQ-39 total change scores (r = 0.25, *p* < 0.05).

Regarding the influence of PD-related medication (LEDD total score, dopamine agonist and non-dopamine agonist subscores of the LEDD, i.e., LEDD total score – dopamine agonist subscore) on patients’ affective symptoms (PDQ-39 emotional well-being SI, HADS depression and anxiety), no significant association was found at baseline. Results of these correlation analyses are also shown in Additional file 1: Table S5. After 6 and 12 months after DBS surgery, change scores of the PDQ-39 emotional well-being subscale did neither significantly correlate with the PD-related medication at baseline nor with changes in this medication (see Table S6). In conclusion, there was little indication for a significant influence of PD-related medication on our patients’ affective symptoms at baseline, 6 or 12 months follow-up.

We then also investigated a potential influence of the LEDD given at baseline, 6- and 12-months follow-up on the QoL at the respective time points. At baseline, the PDQ-39 SI did not significantly correlate with the LEDD (Spearman’s ρ = 0.15, *p* = n.s.). Regarding PDQ-39 subscores, the LEDD significantly correlated with the mobility subscore (Spearman’s ρ = 0.21, *p* < 0.05). At 6- and 12-months follow-up, the PDQ-39 summary index significantly correlated with the LEDD at the respective time points (Spearman’s ρ = 0.26, *p* < 0.01 and Spearman’s ρ = 0.30, *p* < 0.05). At both time points, patients with worse QoL (i.e., higher PDQ-39 scores) had higher LEDD dosages. This effect in the PDQ-39 summary index was mostly driven by the mobility, stigma and cognition subscales at 6 months follow-up (Spearman’s ρ = 0.22–0.26, all *p* < 0.05) and by the ADL and communication subscales at 12 months follow-up (Spearman’s ρ = 0.27–0.28, *p* < 0.05).

### Multiple linear regression analyses

#### Prediction of quality of life at baseline

**Fig. 2:**
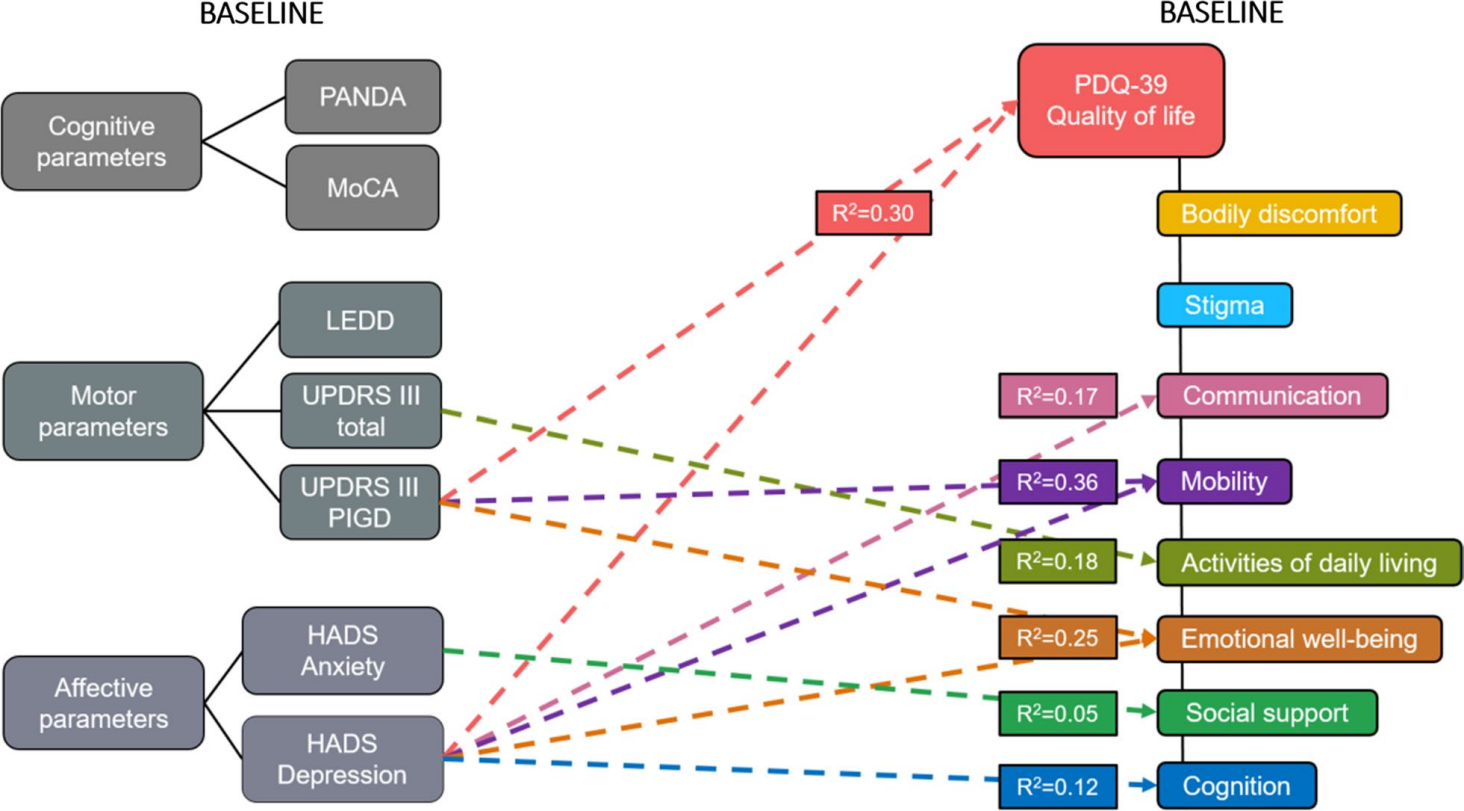
Prediction of the PDQ-39 scales at baseline by cognitive, motor and affective baseline parameters. PDQ-39 total was predicted by the UPDRS-III PIGD subscore (β = 1.18, *p* = 0.003) and HADS depression (β = 0.59, *p* < 0.001), accounting for 30% of the variance [F(3,86) = 13.64, *p* < 0.001]. The PDQ-39 subscale communication was predicted by the HADS depression (β = 0.71, *p* = 0.008), accounting for 17% of the variance [F(2,87) = 10.05, *p* < 0.001]. The PDQ-39 subscale mobility was predicted by the UPDRS-III PIGD subscore (β = 3.40, *p* < 0.001) and HADS depression (β = 0.99, *p* = 0.001), which accounted for 36% of the variance [F(5,84) = 11.19, *p* < 0.001]. The PDQ-39 subscale ADL was predicted by the UPDRS-III total score (β = 0.68, *p* = 0.003) and accounted for 18% of the variance [F(4,85) = 5.93, *p* < 0.001]. The PDQ-39 subscale emotional well-being was predicted by the UPDRS-III PIGD subscore (β = 1.20, *p* = 0.02) and HADS depression (β = 0.80, *p* = 0.001), accounting for 25% of the variance [F(3,86) = 10.94, *p* < 0.001]. The PDQ-39 subscale social support was predicted by the HADS anxiety (β = 0.27, *p* = 0.03), explaining 4% of the variance [F(1,88) = 4.64, *p* = 0.03]. The PDQ-39 subscale cognition was predicted by the HADS depression (β = 0.60, *p* = 0.014), explaining 12% of the variance [F(2,87) = 7.26, *p* = 0.001]. For clarity, only significant predictors from the final regression model are displayed. Each colour of the arrows represents a separate multiple linear regression model

For clarity, details on the respective explained variances for each multiple regression equation are documented in (the legend of) Fig. 2. The baseline UPDRS-III total score medOFF significantly predicted the absolute baseline score of the PDQ-39 subscale ADL (β = 0.68, *p* = 0.003). Furthermore, the baseline UPDRS-III PIGD score medOFF significantly predicted the baseline PDQ-39 total score (β = 1.18, *p* = 0.003) as well as the subscales mobility (β = 3.40, *p* < 0.001) and emotional well-being (β = 1.20, *p* = 0.02). The baseline HADS anxiety scores significantly predicted the absolute baseline scores of the PDQ-39 subscale social support (β = 0.27, *p* = 0.03). Lastly, the HADS depression baseline scores significantly predicted the absolute PDQ-39 total score (β = 0.59, *p* < 0.001), as well as the subscales communication (β = 0.71, *p* = 0.008), mobility (β = 0.99, *p* = 0.001), emotional well-being (β = 0.80, *p* = 0.001), and cognition (β = 0.60, *p* = 0.014) at baseline.

#### Prediction of changes in quality of life at 6 months follow-up

The baseline LEDD significantly predicted changes in the QoL stigma subscale (β = −0.14, *p* = 0.003) at 6 months after the DBS surgery and explained 16% of variance in the data [F(3,86) = 6.52, *p* < 0.001]. The baseline UPDRS-III PIGD score medOFF significantly predicted changes in the QoL subscale mobility (β = 1.60, *p* = 0.039) at 6 months after the DBS surgery and explained 5% of the variance in the data [F(1,88) = 4.39, *p* = 0.039]. Furthermore, the baseline HADS depression scores significantly predicted changes in the PDQ-39 total score (β = 0.50, *p* = 0.003) at 6 months after the DBS surgery and explained 10% of the variance in the data [F(1,88) = 9.62, *p* = 0.003].

#### Prediction of changes in quality of life at 12 months follow-up

**Fig. 3:**
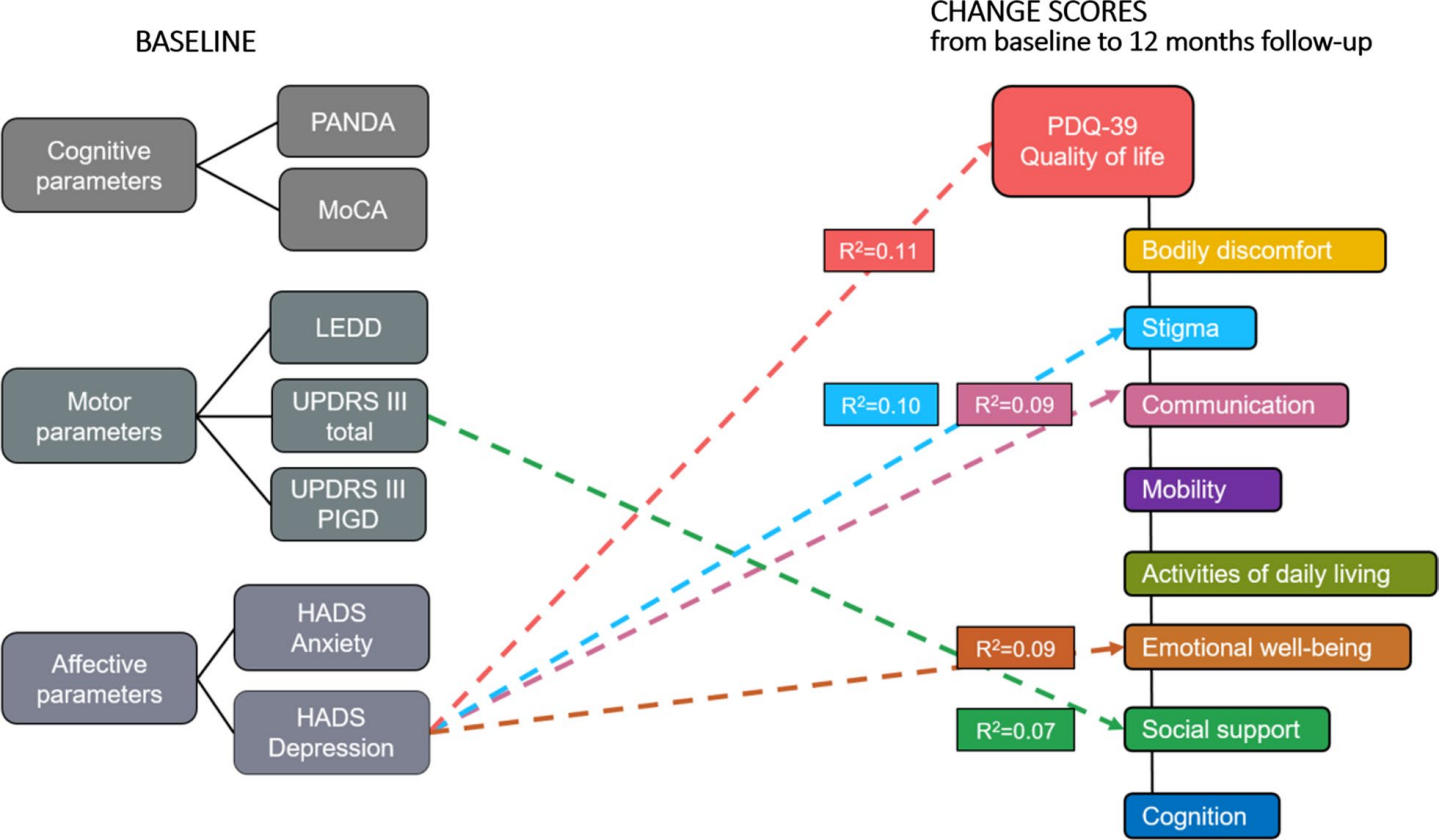
– Prediction of the PDQ-39 change scores at 12 months follow-up by pre-operative baseline parameters. Change scores in the PDQ-39 total score were predicted by the HADS depression (β = 0.52, *p* = 0.007), accounting for 11% of the variance [F(1,61) = 7.82, *p* = 0.007]. Change scores in the PDQ-39 subscale stigma were predicted by the HADS depression (β = 0.73, *p* = 0.014), explaining 10% of the variance [F(1,61) = 6.42, *p* = 0.014]. Change scores in the PDQ-39 subscale communication were predicted by the HADS depression (β = 0.77, *p* = 0.019), accounting for 9% the variance each [F(1,61) = 5.76, *p* = 0.019]. Change scores in the PDQ-39 subscale emotional well-being were predicted by the HADS depression (β = 0.74, *p* = 0.015), explaining 9% of the variance [F(1,61) = 6.33, *p* = 0.015]. Change scores in the PDQ-39 subscale social support were predicted by the UPDRS-III total score (β = 0.26, *p* = 0.037), explaining 7% of the variance [F(1,61) = 4.56, *p* = 0.037]. For clarity, only significant predictors from the final regression model are displayed. Each colour of the arrows represents a separate multiple linear regression. *HADS* Hospital Anxiety and Depression Scale, *MoCA* Montreal Cognitive Assessment, *PANDA* Parkinson’s Neuropsychometric Dementia Assessment, *PDQ-39* Parkinson’s Disease Questionnaire 39, *R*^2^ bias corrected regression coefficient R^2^, *UPDRS-III* Unified Parkinson’s Disease Rating Scale Part III, *UPDRS-III PIGD* postural instability and gait disorder subscore of the Unified Parkinson’s Disease Rating Scale Part III

As can be seen in Fig. 3, the baseline UPDRS III total score medOFF significantly predicted the QoL subscale social support (β = 0.26, *p* = 0.037). Also, scores in the HADS depression scale at baseline significantly predicted the PDQ-39 total score (β = 0.52, *p* = 0.007), and its three subscales stigma (β = 0.73, *p* = 0.014), communication (β = 0.77, *p* = 0.019), and emotional well-being (β = 0.74, *p* = 0.015). For details on the respective explained variances of the regression equations, see Fig. 3.

In order to control for potential confounding effects by comorbidities, all 90 patients were screened for potentially QoL-affecting diagnoses between baseline and 12 months follow-up assessment. Fourteen patients were identified. Therefore, the regression analyses were repeated with the subgroup of 76 patients (baseline and 6 months follow-up, previously 90 patients) and 54 patients (12 months follow-up, previously 63 patients). The overall pattern of the results remained the same for this restricted analysis. These complementary results are depicted in Additional file 1: Figure S1.

### Potential effects of gender on the regression models

The moderation analyses did not reveal any significant moderation by gender on the prediction effects between the baseline predictors and QoL or changes in QoL at baseline, 6- or 12-months follow-up.

## Discussion

This study investigated the impact of motor, cognitive, and affective symptoms on predicting QoL in PD patients up to 12 months after DBS surgery. Following DBS surgery, significant improvements were observed for the overall QoL (as assessed by the PDQ-39) and multiple subscales of the PDQ-39 from baseline to 6 and 12 months. Notably, the improved PDQ-39 total score represented meaningful clinical changes [26] comparable with previous studies [6]. The findings of more pronounced improvements in the PDQ-39 subdomains mobility, ADL, bodily discomfort, and stigma (relative to the other subdomains) are consistent with previous reports [6, 7, 11, 27] and can thus be considered a reliable outcome of the STN-DBS. While the cognitive performance before STN-DBS had no predictive power, QoL at baseline and improvements in QoL after STN-DBS were predicted by motor and affective symptoms at baseline. In particular, worse QoL at baseline was shown for PD patients with worse objective motor scores, worse postural instability and gait disorder (operationalised by the PIGD score), and worse symptoms of anxiety and depression at baseline. At 6 months after DBS surgery, more significant improvements in QoL were shown for PD patients with lower LEDD, higher scores in the HADS depression scale and worse postural instability and gait disorder (i.e., the PIGD score) at baseline. Concerning the QoL at 12 months after the DBS surgery, more significant improvements were shown for PD patients with worse objective motor symptoms and those with worse depressive symptoms at baseline.

The current study is the first to comprehensively explore the prediction of subdomains of QoL by motor, cognitive, and affective baseline parameters in PD patients undergoing STN-DBS. The comprehensive study design allowed us to evaluate the specific contribution of a given predictor parameter on a PDQ-39 subdomain, even if this parameter was not predictive of the PDQ-39 total score. For instance, UPDRS-III motor scores specifically predicted the QoL subdomain ADL at baseline and the QoL subdomain social support at 12 months follow-up but did not predict overall QoL at these two time points. Moreover, the current analyses emphasise that the prediction of QoL by baseline motor and affective parameters depends on the time point of the QoL assessment after STN-DBS. These results may well explain the ambiguous findings in the literature regarding baseline predictors for postoperative QoL in PD patients [6, 12, 27]. In the current patient sample, LEDD, motor performance or the PIGD score at baseline were predictive of QoL at specific time points before or after the STN-DBS surgery. Similarly, anxiety was predictive of QoL only at baseline, whereas depression predicted QoL at all three time points (baseline, 6, and 12 months follow-ups).

As in previous studies [6, 9, 12, 27], cognitive baseline performance did neither predict QoL before nor after STN-DBS surgery in the current sample of PD patients. A likely explanation for this consistent finding across studies lies in the selection criteria for the STN-DBS in PD. PD patients who are candidates for STN-DBS are commonly screened for cognitive deficits and the surgery is only performed in those who do not exhibit a relevant cognitive impairment. Thus, PD patients undergoing STN-DBS (including the current sample) show good and less variable cognitive performance compared to a comparable sample of patients with PD who were not selected for DBS surgery [28].

Subclinical depressive symptoms at baseline seem to have beneficial short- and long-term effects on overall QoL and multiple QoL subdomains within the first 12 months after the STN-DBS surgery. This finding is of clinical relevance since severe depression is commonly assumed to constitute a contra-indication for PD patients to receive DBS as it is associated with an increased risk of suicide within the first year after surgery [29]. Our data show that PD patients with subclinical depressive symptoms are eligible for STN-DBS since higher subclinical depressive symptoms at baseline were associated with better QoL outcomes after DBS surgery. The observed longitudinal effect of subclinical depressive symptoms on self-reported QoL might result from the interaction between symptoms of depression, motor symptoms, and QoL. STN-DBS surgery primarily aims at reducing motor symptoms, and previous studies have shown that postoperative motor improvement significantly correlates with QoL improvements [30]. However, this effect could be mediated by changes in symptoms of depression [30]. That depressive symptoms mediate the effect of motor symptoms on (postoperative) QoL may result from the patients’ psychological burden caused by their intense preoperative motor symptoms resulting in symptoms of depression. Therefore, greater improvement in QoL after STN-DBS surgery would be predicted by both more pronounced depressive and motor symptoms at the (preoperative) baseline, which was also shown by the current study outcome. Future studies should further elucidate the intimate relationship between preoperative motor and depressive symptoms in PD patients and their impact on the outcome after STN-DBS surgery.

Some limitations to this study can be acknowledged. Due to the study’s retrospective nature, there was no prospective selection of suitable parameters; only the parameters already recorded within the clinical framework of the preoperative assessment of the PD patients undergoing STN-DBS could be used. However, it should be mentioned that the current selection of clinical assessments, neuropsychological tests, and questionnaires applied in the preoperative assessment for DBS in PD follows established guidelines. For instance, the PDQ-39 is a well-established, disease-specific questionnaire to assess quality of life in PD patients [5, 6], the MoCA and PANDA have been shown to be sensitive measures to assess cognitive performance in PD [17, 18], and the UPDRS-III is a standard measure in clinical practice to assess the severity of motor symptoms in PD patients [6, 9, 12]. However, the relatively small variance in QoL after STN-DBS explained by baseline parameters (5–17%) raises the consideration of other baseline predictors for QoL development after DBS surgery in patients with PD. Notably, non-motor symptoms of PD, e.g., pain, bladder voiding, and concentration deficits, as well as being less affected by fainting, have previously been identified as baseline predictors for QoL improvements after STN-DBS [10].

Furthermore, the current statistical power was insufficient to assess the potential effects of the different subdomains of the applied cognitive tests, such as short-term memory, executive or visuospatial functions, on QoL in PD patients with STN-DBS (rather than using the total scores of the cognitive tests). Moreover, a growing number of studies indicate a relevant prevalence of subjective cognitive decline (SCD) in PD patients, which refers to a subjectively perceived decrease in cognitive performance despite an unremarkable performance in formal cognitive testing [31]. Thus, future studies should explore SCD as a potential cognitive predictor for QoL in patients with PD after STN-DBS.

Future studies should also include a baseline assessment of apathy since previous studies suggest that higher apathy scores in PD patients before DBS surgery are associated with less favourable QoL outcomes [32]. Moreover, the patients’ expectations concerning the DBS surgery should be evaluated since apathy and depressive symptoms at baseline predict a subjectively perceived negative postoperative outcome [32]. However, a larger sample than in the current study is needed to increase the number of candidate predictors and to include the above mentioned parameters without reducing the statistical power and, therefore, the results’ validity.

## Conclusion

To conclude, this study highlights the importance of preoperatively assessing multiple motor and affective parameters in PD patients who are potential candidates for STN-DBS. Based on this assessment, a longitudinal prediction of both overall QoL and QoL subdomains after STN-DBS can be formulated to support the decision-making process of a PD patient’s eligibility for receiving DBS surgery. Therefore, this study adds to the overarching goal of further optimising the work-up of PD patients undergoing STN-DBS surgery.

### Supplementary Information

**Additional file 1.** Supplementary material.

## Data Availability

The datasets used and/or analysed during the current study are available from the corresponding author on reasonable request.

## Abbreviations

ADL: Activities of daily living
HADS: Hospital Anxiety and Depression Scale
H&Y: Hoehn and Yahr scale
LEDD: Levodopa equivalent daily dose
MoCA: Montreal Cognitive Assessment
PANDA: Parkinson’s Neuropsychometric Dementia Assessment
PD: Parkinson’s disease
PDQ-39 SI: Parkinson’s Disease Questionnaire 39 Summary Index
QoL: Quality of life
STN-DBS: Deep brain stimulation of the subthalamic nucleus
UPDRS-III medOFF: Unified Parkinson’s Disease Rating Scale Part III assessed without PD-related medication
UPDRS-III medOFF PIGD: Postural instability and gait disorder subscore calculated from item 27–30 of the Unified Parkinson’s Disease Rating Scale Part III assessed without PD-related medication
