# Supplementary material for: Preoperative motor deficits and depressive symptoms predict quality of life in patients with Parkinson’s disease at different time points after surgery for subthalamic stimulation: a retrospective study
Source: Neurol Res Pract. 2024 Feb 8;6:8. doi: 10.1186/s42466-023-00303-2 (PMC10851535; doi:10.1186/s42466-023-00303-2)
Supplement: Supplementary file 1 — Additional file 1. Supplementary material. [file 42466_2023_303_MOESM1_ESM.pdf]

Supplementary material for the manuscript "*Preoperative motor deficits and depressive symptoms predict quality of life in patients with Parkinson's disease at different time points after surgery for subthalamic stimulation: a retrospective study*" by Carolin Semmler et al.

Table S1. Medication of PD patients at baseline.

Medication group	*Active substance*	*N*	*Min daily dosage*	*Max daily dosage*	*Mean*	*Std*
Anti-depressants	Agomelatin	1	25.0	25.0	25.0	-
	Citalopram	2	10.0	10.0	10.0	0.0
	Selegilin	3	10.0	10.0	10.0	0.0
	Venlafaxin	2	37.5	150.0	93.8	79,6
	Duloxetin	1	60.0	60.0	60.0	-
	Mirtazapin	3	7.5	30.0	17.5	11.5
NMDA antagonists	Amantadin	29	50.0	450.0	194.8	87.0
Benzo-diazepines	*Clonazepam*	*3*	*0.1*	*0.5*	*0.4*	*0.2*
	Bromazepam	*1*	*1.5*	*1.5*	*1.5*	-
Dopamine agonists	*Piribedil*	*16*	*50.0*	*200.0*	*150.0*	*51.6*
	Ropinirol	*14*	*2.0*	*22.0*	*12.7*	*4.8*
	Rotigotin	*19*	*1.0*	*16.0*	*7.6*	*4.1*
	Pramipexol	*40*	*0.2*	*12.0*	*2.5*	*1.8*
	Apomorphin	*1*	*90.0*	*90.0*	*90.0*	-
	Cabergolin	*1*	*5.0*	*5.0*	*5.0*	-

Table S2. Results of correlation analyses for QoL prediction at baseline.

	PANDA total	MoCA total	LEDD	UPDRS-III total medOFF	UPDRS-III PIGD medOFF	HADS anxiety T-value	HADS depression T-value
PDQ-39 total SI	-0.15	-0.09	0,15	**0,22 ***	**0,35 *****	**0,43 *****	**0,43 *****
PDQ-39 mobility SI	-0.09	-0,08	**0,21 ***	**0,34 *****	**0,56 *****	**0,26 ***	**0,34 *****
PDQ-39 ADL SI	0.00	-0,01	0,08	**0,39 *****	**0,32 ****	**0,21 ***	**0,23 ***
PDQ-39 emotional well-being SI	-0.10	-0,02	0,16	0,05	**0,25 ***	**0,40 *****	**0,47 *****
PDQ-39 stigma SI	**-0.22 ***	-0,01	-0,15	0,11	0,09	**0,36 *****	**0,40 *****
PDQ-39 social support SI	-0.18	-0,04	0,15	0,03	0,16	**0,26 ***	0,15
PDQ-39 cognition SI	-0.12	-0,18	0,20	0,02	0,17	**0,33 *****	**0,38 *****
PDQ-39 communication SI	-0.14	-0,04	0,11	0,10	0,12	**0,43 *****	**0,43 *****
PDQ-39 bodily discomfort SI	-0.04	-0,11	0,10	0,12	0,13	**0,34 *****	**0,28 ****

The correlation coefficients for the respective correlations between the PDQ parameters at baseline in the first column and the cognitive, motor, or affective parameters at baseline in the first row. Significant correlations are marked in bold. HADS = Hospital Anxiety and Depression Scale, LEDD = Levodopa equivalent daily dose, MoCA = Montreal Cognitive Assessment, PANDA = Parkinson's Neuropsychometric Dementia Assessment, PDQ-39 SI = Parkinson's Disease Questionnaire 39 Summary Index, UPDRS-III medOFF = Unified Parkinson's Disease Rating Scale Part III assessed without PD-related medication, UPDRS-III medOFF PIGD = postural instability and gait disorder subscore calculated from item 27 - 30 of the Unified Parkinson's Disease Rating Scale Part III assessed without PD-related medication. Significance levels are indicated by asterisks: * = $p < 0.05$, ** = $p < 0.01$, *** = $p < 0.001$.

Table S3. Results of correlation analyses for QoL change scores at 6 months follow-up.

	PANDA total	MoCA total	LEDD	UPDRS-III total medOFF	UPDRS-III PIGD medOFF	HADS anxiety T-value	HADS depression T-value
PDQ-39 total SI	0,01	0,14	-0,08	0,14	0,06	0,17	**0,26 ***
PDQ-39 mobility SI	0,00	0,06	0,12	0,10	**0,24 ***	0,10	0,20
PDQ-39 ADL SI	0,01	0,19	-0,09	0,17	-0,02	0,02	0,15
PDQ-39 emotional well-being SI	0,09	0,09	-0,09	0,05	-0,08	0,08	0,18
PDQ-39 stigma SI	-0,06	0,16	**-0,23 ***	-0,06	-0,13	**0,24 ***	**0,27 ***
PDQ-39 social support SI	0,02	0,05	-0,10	0,08	0,02	0,15	0,04
PDQ-39 cognition SI	0,04	0,09	-0,07	0,02	0,00	0,04	0,17
PDQ-39 communication SI	0,01	0,13	0,05	0,03	0,04	0,19	0,18
PDQ-39 bodily discomfort SI	-0,02	-0,10	0,00	0,18	0,02	0,11	0,15

The correlation coefficients for the respective correlations between the PDQ change parameters at 6 months follow-up in the first column and the cognitive, motor, or affective parameters at baseline in the first row. Significant correlations are marked in bold. HADS = Hospital Anxiety and Depression Scale, LEDD = Levodopa equivalent daily dose, MoCA = Montreal Cognitive Assessment, PANDA = Parkinson's Neuropsychometric Dementia Assessment, PDQ-39 SI = Parkinson's Disease Questionnaire 39 Summary Index, UPDRS-III medOFF = Unified Parkinson's Disease Rating Scale Part III assessed without PD-related medication, UPDRS-III medOFF PIGD = postural instability and gait disorder subscore calculated from item 27 - 30 of the Unified Parkinson's Disease Rating Scale Part III assessed without PD-related medication. Significance levels are indicated by asterisks: * = $p < 0.05$, ** = $p < 0.01$, *** = $p < 0.001$.

Table S4. Results of correlation analyses for QoL change scores at 12 months follow-up.

	PANDA total	MoCA total	LEDD	UPDRS-III total medOFF	UPDRS-III PIGD medOFF	HADS anxiety T-value	HADS depression T-value
PDQ-39 total SI	-0,01	0,11	0,08	0,03	0,17	0,09	**0,25** *
PDQ-39 mobility SI	-0,18	-0,07	0,18	0,02	0,17	0,14	0,25
PDQ-39 ADL SI	-0,03	0,08	0,10	0,16	0,13	0,04	0,11
PDQ-39 emotional well-being SI	0,02	0,21	0,05	-0,19	-0,10	0,16	**0,29** *
PDQ-39 stigma SI	-0,01	0,19	-0,13	0,04	0,05	0,16	**0,27** *
PDQ-39 social support SI	0,09	0,13	0,03	**0,28** *	0,21	0,16	0,07
PDQ-39 cognition SI	-0,08	0,01	0,16	-0,10	0,09	-0,15	0,09
PDQ-39 communication SI	0,07	0,09	0,14	-0,03	0,15	0,21	**0,32** *
PDQ-39 bodily discomfort SI	-0,01	-0,08	-0,11	0,09	0,04	0,08	0,15

The correlation coefficients for the respective correlations between the PDQ change parameters at 12 months follow-up in the first column and the cognitive, motor, or affective parameters at baseline in the first row. Significant correlations are marked in bold. HADS = Hospital Anxiety and Depression Scale, LEDD = Levodopa equivalent daily dose, MoCA = Montreal Cognitive Assessment, PANDA = Parkinson's Neuropsychometric Dementia Assessment, PDQ-39 SI = Parkinson's Disease Questionnaire 39 Summary Index, UPDRS-III medOFF = Unified Parkinson's Disease Rating Scale Part III assessed without PD-related medication, UPDRS-III medOFF PIGD = postural instability and gait disorder subscore calculated from item 27 - 30 of the Unified Parkinson's Disease Rating Scale Part III assessed without PD-related medication. Significance levels are indicated by asterisks: * = $p < 0.05$, ** = $p < 0.01$, *** = $p < 0.001$.

Table S5. Results of correlation analyses for QoL at baseline and dopaminergic medication at baseline.

Quality of life	LEDD	Dopamine agonists	LEDD rest
PDQ-39 total SI	0.15	-0.17	**0.22 ***
PDQ-39 mobility SI	**0.21 ***	-0.13	**0.24 ***
PDQ-39 ADL SI	0.08	-0.20	0.12
PDQ-39 emotional well-being SI	0.16	-0.02	0.17
PDQ-39 stigma SI	-0.15	**-0.27 ***	-0.06
PDQ-39 social support SI	0.15	-0.02	**0.21 ***
PDQ-39 cognition SI	0.20	-0.09	0.20
PDQ-39 communication SI	0.11	0.01	0.15
PDQ-39 bodily discomfort SI	0.10	-0.15	0.14
HADS anxiety t-value	0.00	-0.13	0.03
HADS depression t-value	0.03	-0.14	0.04

Table S6A. Results of correlation analyses for change scores of QoL at 6-months follow-up and dopaminergic medication at baseline and for change scores at 6 months follow-up.

Change scores Baseline – 6 months follow-up	Baseline			Change scores Baseline – 6 months follow-up		
Quality of life	LEDD	Dopamine agonists	LEDD rest	LEDD	Dopamine agonists	LEDD rest
PDQ-39 total SI	-0,08	-0,19	-0,06	-0,09	-0,02	-0,05
PDQ-39 mobility SI	0,12	-0,01	-0,04	**-0,29 ****	**-0,22 ***	-0,16
PDQ-39 ADL SI	-0,09	**-0,21 ***	-0,06	-0,07	-0,04	-0,03
PDQ-39 emotional well-being SI	-0,09	-0,11	-0,09	-0,03	0,08	-0,03
PDQ-39 stigma SI	**-0,23 ***	**-0,25 ***	-0,17	0,03	0,03	0,04
PDQ-39 social support SI	-0,10	-0,16	-0,06	0,06	0,02	0,07
PDQ-39 cognition SI	-0,07	-0,04	-0,11	-0,14	-0,07	-0,09
PDQ-39 communication SI	0,05	-0,14	0,12	0,01	0,14	-0,05
PDQ-39 bodily discomfort SI	0,00	-0,13	0,00	-0,08	0,00	-0,04

Table S6B. Results of correlation analyses for change scores of QoL at 12-months follow-up and dopaminergic medication at baseline and for change scores at 12 months follow-up.

Change scores Baseline – 12 months follow-up	Baseline			Change scores Baseline – 12 months follow-up		
Quality of life	LEDD	Dopamine agonists	LEDD rest	LEDD	Dopamine agonists	LEDD rest
PDQ-39 total SI	0.08	**-0.36 ****	0.12	-0.16	0.22	-0.17
PDQ-39 mobility SI	0.18	-0.21	0.17	-0.13	0.19	-0.12
PDQ-39 ADL SI	0.10	**-0.28 ***	0.16	-0.14	0.18	-0.18
PDQ-39 emotional well-being SI	0.05	-0.17	0.06	-0.05	0.23	-0.10
PDQ-39 stigma SI	-0.13	**-0.31 ***	-0.06	-0.16	0.07	-0.15
PDQ-39 social support SI	0.03	-0.21	0.05	-0.17	-0.11	-0.07
PDQ-39 cognition SI	0.16	0.00	0.15	-0.17	0.07	-0.17
PDQ-39 communication SI	0.14	-0.07	0.14	-0.11	0.07	-0.10
PDQ-39 bodily discomfort SI	-0.11	**-0.33 ****	-0.03	0.00	**0.32 ***	-0.04

Figure S1 A-B – Prediction of the PDQ-39 at baseline (a) and PDQ-39 change scores at 6 months (b) and 12 months follow-up (c) by pre-operative baseline parameters after exclusion of 14 patients with QoL-relevant health events between DBS surgery and 12 months follow-up assessment (n = 76 and n = 54, respectively).

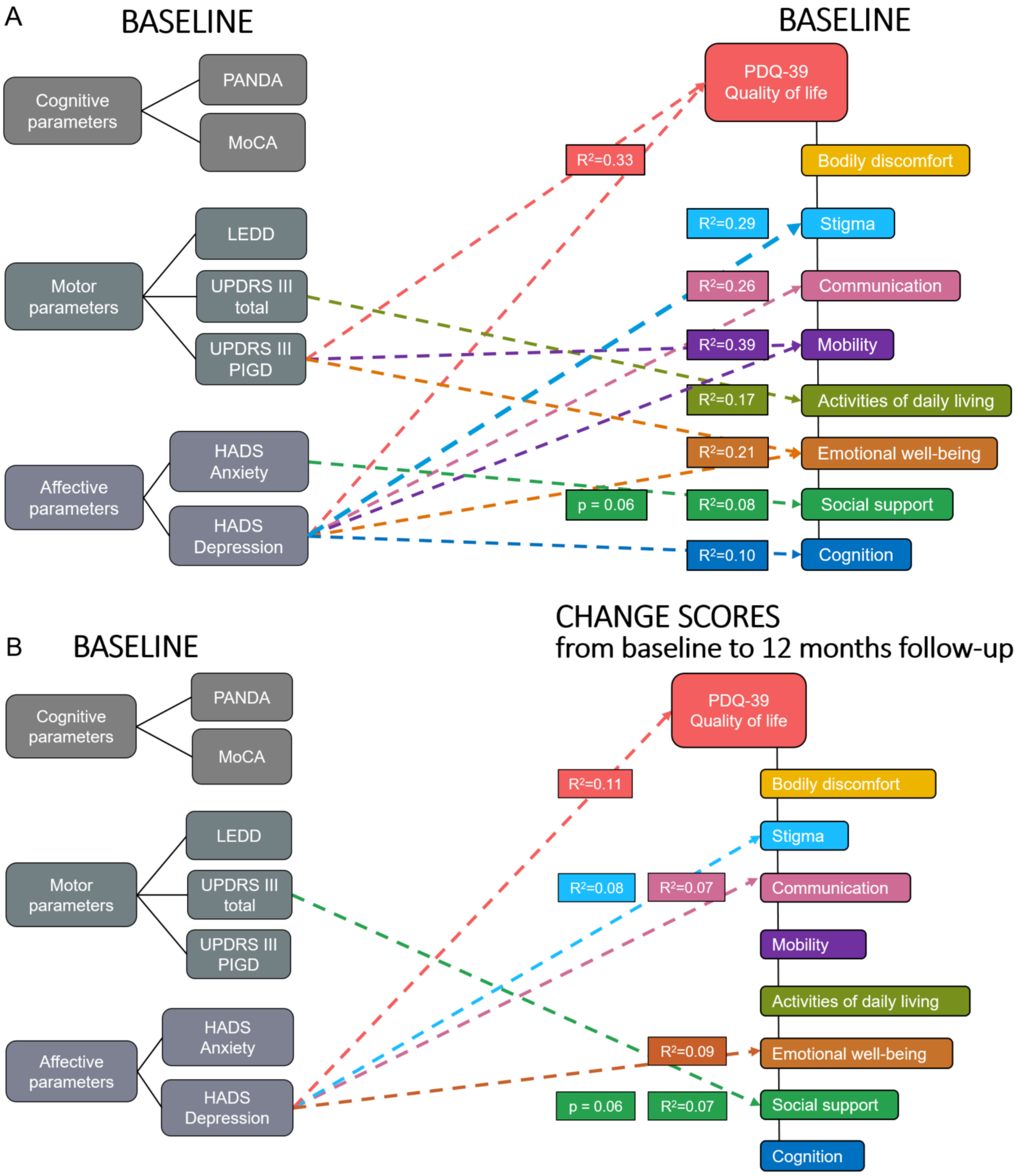

Note: The regression analyses were repeated after excluding 14 patients with comorbidities that might have influenced the QoL of the PD patients. Notably, the overall pattern of the results remained the same, while the variance explained in the regression models was reduced due to the lower number of PD patients. At baseline, the prediction of the PDQ-39 social support subscale by the HADS anxiety became a trend (p = 0.06). For the prediction of changes in QoL after 6 months after DBS surgery, the prediction of the PDQ-39 mobility subscale by the baseline PIGD score was not significant anymore but only

a trend ($p = 0.08$). Additionally, the PDQ-39 stigma subscale could not be predicted anymore by baseline LEDD, but was instead predicted by the HADS depression score ($\beta = 0.80$, $p < 0.05$) and explained 13% of the variance in the data ($F(2,75) = 6.42$, $p < 0.01$). Also, changes in the PDQ-39 communication subscale was significantly predicted by the HADS depression score ($\beta = 0.81$, $p < 0.01$) and explained 10% of the variance in the data ($F(1,75) = 7.95$, $p < 0.01$). At 12 months follow-up, the prediction of the PDQ-39 social support subscale by the UPDRS-III total score became a trend ($p = 0.06$).
